# The Molecular Engineering of an Anti-Idiotypic Antibody for Pharmacokinetic Analysis of a Fully Human Anti-Infective

**DOI:** 10.1371/journal.pone.0145381

**Published:** 2015-12-23

**Authors:** She Yah Lim, Conrad E. Z. Chan, Malgorzata M. Lisowska, Brendon J. Hanson, Paul A. MacAry

**Affiliations:** 1 Department of Microbiology, National University of Singapore, Singapore, Singapore; 2 Immunology Program, Centre for Life Sciences, National University of Singapore, Singapore, Singapore; 3 NUS Graduate School for Integrative Sciences and Engineering, Singapore, Singapore; 4 Defence Medical and Environmental Research Institute, DSO National Laboratories, Singapore, Singapore; Tulane University, UNITED STATES

## Abstract

Anti-idiotype monoclonal antibodies represent a class of reagents that are potentially optimal for analyzing the pharmacokinetics of fully human, anti-infective antibodies that have been developed as therapeutic candidates. This is particularly important where direct pathogen binding assays are complicated by requirements for biosafety level III or IV for pathogen handling. In this study, we describe the development of a recombinant, anti-idiotype monoclonal antibody termed E1 for the detection of a fully human, serotype-specific, therapeutic antibody candidate for the BSLIII pathogen Dengue virus termed 14c10 hG1. E1 was generated by naïve human Fab phage library panning technology and subsequently engineered as a monoclonal antibody. We show that E1 is highly specific for the fully-folded form of 14c10 hG1 and can be employed for the detection of this antibody in healthy human subjects’ serum by enzyme linked immunosorbent assay. In addition, we show that E1 is capable of blocking the binding of 14c10 hG1 to dengue virus serotype 1. Finally, we show that E1 can detect 14c10 hG1 in mouse serum after the administration of the therapeutic antibody *in vivo*. E1 represents an important new form of ancillary reagent that can be utilized in the clinical development of a therapeutic human antibody candidate.

## Introduction

Dengue is the world’s most prevalent mosquito-borne viral disease. Annual estimates of 390 million infections have been reported to occur across around 100 countries, with 2.5 billion people living in dengue endemic countries. Dengue remains as a major and significant clinical problem and the burden should be reduced with vaccination. However, there is currently no vaccine or antiviral therapy available [1, 2]. The only form of supportive treatment is fluid management and prophylactic platelet transfusions. However, the latter is not the standard of care [3].

Dengue virus (DENV) is a biosafety level III pathogen in Europe and North America and comprises four distinct serotypes (DENV-1 to DENV-4) [4]. Recently, a neutralising fully human monoclonal antibody (14c10 hG1) specific for DENV-1 had been characterised. 14c10 hG1 was isolated from a convalescent DENV-1 infected patient. It was shown in *in vivo* studies that 14c10 hG1 exhibited antiviral activity at picomolar concentrations by inhibiting both the virus attachment step and post attachment step. This study showed that 14c10 hG1 is a potentially good therapeutic candidate for the treatment of DENV-1 infection [5] but clinical development is complicated by the high biosafety level rating of this pathogen in sites where Dengue is not endemic.

Pharmacokinetics (PK) is an important parameter for the preclinical and clinical development of therapeutics drugs and antibodies [6]. Assays that underlie the analysis of the pharmacokinetics of therapeutic antibodies should be sensitive, precise and reproducible, and the extent of these requirements depends on the purpose of the assay (preclinical or clinical) and the phase of drug development [7]. Anti-idiotype antibodies represent useful reagents that can be employed for pharmacokinetic analysis of antibody therapeutics [8].

Antibody phage display is based on the use of filamentous phage which replicates in *Escherichia coli*. DNA encoding the Fab domains are cloned into the phage genome and fused to the phage coat protein. This allows the expression of the Fab on the coat protein of the filamentous phage [9–12]. Here we describe the isolation, molecular engineering, characterization and employment of an anti-idiotypic antibody (E1) targeting a fully human therapeutic antibody candidate (14c10 hG1) from a naïve human Fab phage display library HX01 (Humanyx Pte Ltd, Singapore).

## Materials and Methods

### Cells

Vero cells (ATCC) were maintained in medium 199 (M199) (Invitrogen, USA) supplemented with 8% fetal bovine serum (FBS) (GIBCO, Invitrogen), 1% sodium pyruvate, 1% MEM non-essential amino acid (NEAA) (GIBCO, Invitrogen), in 37°C incubator with 5% CO_2_. Human embryonic kidney 293- 6E (HEK293 -6E) cells were cultured in freestyle 17 medium supplemented with glutamax, pluronic acid and geneticin (GIBCO, Invitrogen) and grown in 37°C incubator with 5% CO_2_. Baby hamster kidney cells (ATCC) were grown in RPMI 1640 (Thermo Scientific, USA) supplemented with 10% FBS (Invitrogen, USA), in 37°C incubator with 5% CO_2_.

### Virus

Dengue 1 (DENV-1) strain EHI and Dengue 2 (DENV-2) strain New Guinea C (NGC) were passaged in vero cells and the virus titers were determined by plaque assay with BHK21 cells.

### Phage display panning

Humanyx Fab phage library (naïve human Fab phage library) (Humanyx Pte Ltd, Singapore) was used for the phage display panning process against the target antigen, 14c10 Fab [5]. The library was enriched for positive clones against 14c10 Fab via panning against 20μg of the target antigen coated onto an immunotube (maxisorp tube) (Nunc, Denmark). A total of four rounds of panning were performed. In each round of panning, the pre-coated immunotube and polyethylene glycol (PEG) (Sigma- Aldrich, USA) precipitated Humanyx library stock were first blocked with blocking buffer (pre-blocked). The pre-blocked PEG precipitated Humanyx library stock was then added to the immunotube, and incubated for 1.5hours at room temperature. In the first round of panning, the immunotube was washed three times with MPBS/T (skim milk prepared in phosphate buffered saline (PBS) containing 0.05% Tween-20), three times of PBS/T and two times of PBS (containing 137mM sodium choride, 2.7mM potassium chloride and 12mM phosphate buffer) (Axil Scientific, Singapore). For subsequent rounds of panning, the immunotube was washed seven times with MPBS/T, seven times of PBS/T and two times of PBS. The phage was then eluted from the immunotube with trypsin protease (Worthington, USA) and used for re-infection of TG1 at log phase growth. The phage was then rescued with helper phage, M13KO7, at an MOI of 20 and plated out onto 15cm 2YTAK agar plates (contained 100μg/ml ampicillin and 25μg/ml kanamycin. Phage was recovered from the agar plates the next day by scraping the bacterial lawn off the 2YTAK agar plates and PEG precipitated with PEG6000 (Sigma- Aldrich, USA). The PEG precipitated phage was used for the subsequent rounds of panning as described above.

### Polyclonal phage ELISA

The pre-blocked PEG precipitated polyclonal phages from the four rounds of panning were tested against the target antigen, 14c10 Fab plus other irrelevant control antigens (D29 Fab and 3H5 Fab) [13] to check for their specificity. The wells were coated with 20μg/ml of the antigens and 4% skim milk in PBS (MPBS) (blocking buffer) (Sigma- Aldrich, USA) overnight at 4°C and blocked with 4% MPBS for 1.5 hours at room temperature. The polyclonal PEG precipitated phage was diluted ten times in 4% MPBS and added to the wells (Thermo Scientific, USA) coated with the antigens and incubated for 1 hour at room temperature. Secondary antibody αM13-HRP (GE healthcare, USA) was diluted 1:5000 in 2% MPBS and the reaction was incubated for one hour at room temperature. The binding signal was detected and visualised with TMB substrate (Thermo Scientific, USA) for ten minutes and stopped with 2M sulphuric acid. For all ELISA washing steps, the plates were washed two times with PBS after overnight coating, two times with PBS/T after blocking step. Subsequent steps were washed with PBS/T for four times except before the addition of TMB substrate in which the plates were washed three times with PBS/T and once with PBS.

### Selection of monoclonals and starter culture

TG1 glycerol stock from pan three of polyclonal phage panning was serially diluted 10^3^ to 10^6^ and plated out on 15cm 2YTAG plates (contained 100μg/ml Ampicillin and 2% glucose) and incubated overnight at 33°C. The next day, 66 colonies were picked from 2YTAG agar plate plated with 10^3^ diluted TG1 glycerol stock, and grown in 96-well U bottom plates containing 2YTAG media. This monoclonal starter culture was incubated overnight at 30°C, 700rpm.

### Monoclonal phage ELISA

Bacterial starter culture from above was transferred to new deep well plate (Thermo Scientific, USA) containing 2YTAG media and grown at 37°C till the OD reached 0.5. M13KO7 helper phage (5 x 10^12^ pfu/ml) diluted 1:100 in 2TYAG media was added into each well and grown at 37°C for 1 hour, shaking at 700 to 800rpm. The plate was spun at 4000g for 10 minutes and the supernatant was removed. The pellet was resuspended in 2YTAK media and incubated 30°C overnight, shaking at 700 to 800rpm.

Meanwhile, 96-well maxisorp plates (Thermo Scientific, USA) were coated with 20μg/ml 14c10 Fab (target antigen) and D29 Fab (irrelevant antigen) overnight. The plates were blocked with 4% MPBS for 1.5hours at room temperature the next day. The overnight cultures from the deep well plates were spun down at 4000g for ten minutes and the supernatant was mixed with equal amount of 4% MPBS and added to the pre-coated and pre-blocked wells. The secondary antibody αM13-HRP (GE Healthcare, USA) was diluted 1:5000 in 2% MPBS. All incubation steps were for one hour at room temperature. The binding signal was detected by development of the reactions with TMB substrate (Thermo Scientific, USA) and stopped with 2M sulphuric acid after ten minutes. A control was included in the setup in which the bacterial culture was not added and the secondary antibody used was αhistag-HRP (Novagen, Germany) diluted in 1:2000 in 2% MPBS. This was to check that the antigens were properly coated onto plates. Plate washing steps were the same as polyclonal phage ELISA.

### Sequencing of Fab library clones

The positive clone from the starter culture plate was inoculated in 2YTAG media and grown overnight. Plasmid was extracted via E.Z.N.A plasmid miniprep kit (Omega Bio- Tek, Inc., USA). Plasmid DNA was sequenced with the primers HX01-01F (5’-AGCGGATAACAATTTCACACA-3’) and pCES-1LR (5’-ACAATCCAGCGGCTGCCGTA-3’) for light chain sequence and pCES-1HF (5’-GGCGCGCCAATTCTATTTCAAG-3’) and HX01-01R (5’-TTTGTCGTCTTTCCAGACGTTAGT-3’) for heavy chain.

### Cloning and antibody expression

Heavy and light chains were cloned via BsmBI/ SfiI and SalI/NotI (New England Biolabs, UK) restriction enzyme sites respectively from the library phagemid vector into the different expression vectors. The expression vector contains the framework for expression of antibody into the mouse chimeric IgG2a with human IgG3 human hinge or mouse IgG2a or Fab. The constructs encoding the heavy and light chains were transfected into mammalian HEK293 cells (ATCC) via branched polyethylenimine (PEI) (Sigma- Aldrich, USA). Supernatants were collected six days after transfection and proteins were purified with protein A agarose beads (Thermo Scientific, USA) for IgG and talon beads (Roche, Switzerland) for Fab. Protein concentrations were determined with Coomassie Plus protein assay kit (Pierce, USA) with bovine serum albumin (BSA) as standard according to the protocol. Purity of the antibodies was determined with sodium dodecyl sulphate polyacrylamide gel electrophoresis (SDS-PAGE) (described below).

### Sodium dodecyl sulphate polyacrylamide gel electrophoresis (SDS-PAGE)

Two microgram of each antibody sample was measured and loaded into each lane of the gel. 12% or 8% resolving polyacrylamide gel and 3% stacking gel were used. Samples were loaded at reducing (addition of dithiothreitol (DTT)) and non-reducing conditions (no addition of reducing agent). Electrophoresis was conducted at initial constant 80V till the bands moved into the resolving gel. Then the voltage was increased to 120V. The gel run was stopped when the dye front reached the bottom of the gel. The gels were then stained with Instant BLUE stain (Expedeon, United Kingdom) and destained with distilled water overnight.

### Testing specificity of phage for 14c10 hG1 in serum

The target antigen, human 14c10 IgG1 (14c10 hG1) and other irrelevant antigens (3H5 hG1 and D29 hG1) were coated onto 96-well plates overnight at a starting concentration of 500μg/ml and serially diluted one in two in PBS. The PEG precipitated phage clone E1 was either diluted one in ten in blocking buffer (4% MPBS) or one in ten with diluted human serum (pre-diluted one in four in blocking buffer) and added to the antigen pre- coated wells. The secondary antibody αM13-HRP (GE Healthcare, USA) was diluted 1:5000 in 2% MPBS. All incubation steps lasted for an hour at room temperature. The binding signal was detected by development of the reactions with TMB substrate (Thermo Scientific, USA) and stopped with 1M sulphuric acid after ten minutes. Plate washing steps were the same as polyclonal phage ELISA.

### Comparison of E1 mG2a and E1 mG2a/hG3

The 96- well maxisorp plates (Thermo Scientific, USA) were coated overnight with 5μg/ml of 14c10 hG1. The antibodies, E1 mG2a and E1 mG2a/hG3 were used at a starting concentration of 5μg/ml and serially diluted half log in blocking buffer (4% MPBS). The binding of the antibodies was detected with anti-mouse-IgG-Fc-HRP (1:5000) (Thermo Scientific, USA) and the reaction was developed with TMB substrate and stopped with 1M sulphuric acid. All incubation steps lasted for one hour at room temperature. Plate washing steps were the same as polyclonal phage ELISA.

### Testing the Specificity of E1 for 14c10 hG1

In the direct ELISA assay, the wells were coated with 5μg/ml of the antigens (14c10 hG1, 3H5 hG1, D29 hG1, HuIgG and blocking buffer (4% MPBS). The detector, E1 mG2a/hG3 was used at 1μg/ml. The binding of the antibodies was detected with anti-mouse-IgG-Fc-HRP (Thermo Scientific, USA) and the reaction developed with TMB substrate. The reaction was stopped with 1M sulphuric acid.

In the sandwich ELISA, the wells were coated at a constant concentration of 5μg/ml of E1 mG2a/hG3 overnight. The antigens were added to the coated wells at a starting concentration of 100μg/ml and serially diluted by a half log in blocking buffer (4% MPBS). In a parallel setup, the antigens (at starting concentration of 100μg/ml) were spiked with purified polyclonal IgGs from healthy subjects at a starting concentration of 12mg/ml and serially diluted by half log in blocking buffer.

Sera from 5 healthy subjects were heat inactivated at 56°C for 30 minutes, centrifuged at 10,000g for 20 minutes at 4°C. Supernatant was transferred to a new tube and pooled. For 25ml sera, we added 20ml of 50% slurry resins (75% Protein A + 25% Protein G) (Pierce, USA). The agarose beads were allowed to bind the polyclonal IgGs overnight at 4°C on a tube rotator. The next day, the beads were transferred to a 25ml disposable column and the beads were washed with 50ml of PBS. The IgGs were eluted from the beads with 50ml of IgG elution buffer and desalted with 30kDa amicon tube (Merck Millipore, Germany).

The secondary antibody anti-human-IgG-Fc-HRP (Thermo Scientific, USA) was used at 1:5000 dilution and the reaction was developed with TMB substrate. The reaction was stopped with 1M sulphuric acid. All binding steps lasted for one hour at room temperature. Plate washing steps were the same as polyclonal phage ELISA.

### Specificity of E1 for 14c10 hG1 in human serum

Blood from ten healthy volunteers were taken and centrifuged at 2000g for 15 minutes and the serum was stored in -80°C freezer before use for the subsequent experiments.

In the sandwich ELISA assay format, the wells were coated overnight with 5μg/ml of E1 mG2a/hG3. Neat serum from each volunteer was spiked with a starting concentration of 20μg/ml 14c10 hG1 and serially diluted by half a log in neat serum. 14c10 hG1 in neat serum was serially diluted three fold in blocking buffer (4% MPBS) (from 1 in 3 to 2187). For every row of serially diluted 14c10 hG1 in serum, there is a control well without the addition of 14c10 hG1. The serially diluted 14c10 hG1 in serum was added to the pre-coated wells with E1 mG2a/hG3. Binding of 14c10 hG1 in serum was detected by α-hu-Fc-IgG-HRP (1:5000) (Thermo Scientific, USA). Each incubation step was one hour at room temperature. The reaction was developed with TMB substrate and stopped with 1M sulphuric acid. Plate washing steps were the same as polyclonal phage ELISA.

### Competition binding assay

96-well plates were coated overnight at a constant concentration of 5μg/ml 4G2 mG2a as a capture for DENV-1 EHI strain. The virus (9 x10^7^ pfu/ml) was used at 1 in 10 dilution. The antibodies (14c10 hG1 and D29 hG1) were used at a fixed concentration of 6μg/ml and pre-incubated with E1 Fab or anti-DENV-2 Fab for one hour at room temperature, at a starting concentration of 50μg/ml and serially diluted by half a log in blocking buffer (4% MPBS), at room temperature, before the addition to DENV-1. α-human-IgG-Fc-HRP (1:5000) (Thermo Scientific, USA) was used as a detector. Each step was incubated for one hour at room temperature. The reaction was developed with TMB substrate and stopped with 1M sulphuric acid.

In the control setup, the wells were coated at a constant concentration of 5μg/ml 4G2 mG2a as a capture for DENV-1 EHI strain and DENV-2 NGC strain. DENV-1 and DENV-2 (2.2 x 10^7^ pfu/ml) were used at 1 in 2 and 1 in 100 dilution respectively. The antibodies (14c10 hG1, D29 hG1, anti-DENV-2 Fab) were used at a constant concentration of 6μg/ml. α-human-IgG-Fc-HRP (Thermo Scientific, USA) and α-histag-HRP (1:2000) (Novagen, Germany) (for Fab) were used as a detector. Each step was incubated for one hour at room temperature. The reaction was developed with TMB substrate and stopped with 1M sulphuric acid. Plate washing steps were the same as polyclonal phage ELISA.

### Establishment of therapeutic mouse model

AG129 mice were inoculated subcutaneously with 200μl of a DENV-1 suspension containing 10^7^pfu/ml DENV-1 WP74. Two days post- infection, the mice were treated intraperitoneally with 10μg of 14c10 hG1. The control untreated group was injected with 200μl of sterile PBS. For the measurement of the level of 14c10 hG1 in the mice, blood was taken from the jugular vein at various time points. Both groups consisted of five AG129 mice. This study was approved and the mice were handled strictly according to the National University of Singapore (NUS) Institutional Animal Care and Use Committee recommendations under protocol number 107/10, and in accordance with the National Advisory Committee for Laboratory Animal Research (NACLAR) Guidelines (Guidelines on the Care and Use of Animals for Scientific Purposes). NUS is an AAALAC accredited institution. All procedures and surgery was performed under isoflurane anesthesia, and all efforts were made to minimise suffering.

### Establishment of prophylaxis mouse model

AG129 mice were first treated intraperitoneally with 10μg of 14c10 hG1 per mouse before infection with 200μl DENV-1 WP74 at 10^7^pfu/ml subcutaneously. The control untreated group was injected with 200μl of sterile PBS instead of 14c10 hG1. For the measurement of the level of 14c10 hG1 in the mice, blood was taken from the jugular vein at various time points. Both groups consisted of five AG129 mice.

### Ethics Statement

Written informed consent was obtained from all study participants and all procedures were carried out under an approved protocol from the National University of Singapore Institutional Review Board under the protocol number 06–196 and the clinical investigation was conducted according to the principles in the Declaration of Helsinki.

### Statistical Analysis

All data presented represent an average of at least two independent experiments. Error bars represent the standard error mean (SEM). Two way analysis of variance (ANOVA) was used for the statistical comparison of the signal binding strength of the two subclasses of antibodies (mG2a and mG2a/hG3) using GraphPad Prism 5.

## Results

### Antibody discovery- Screening and selection of clones from phage library panning

**Fig 1:**
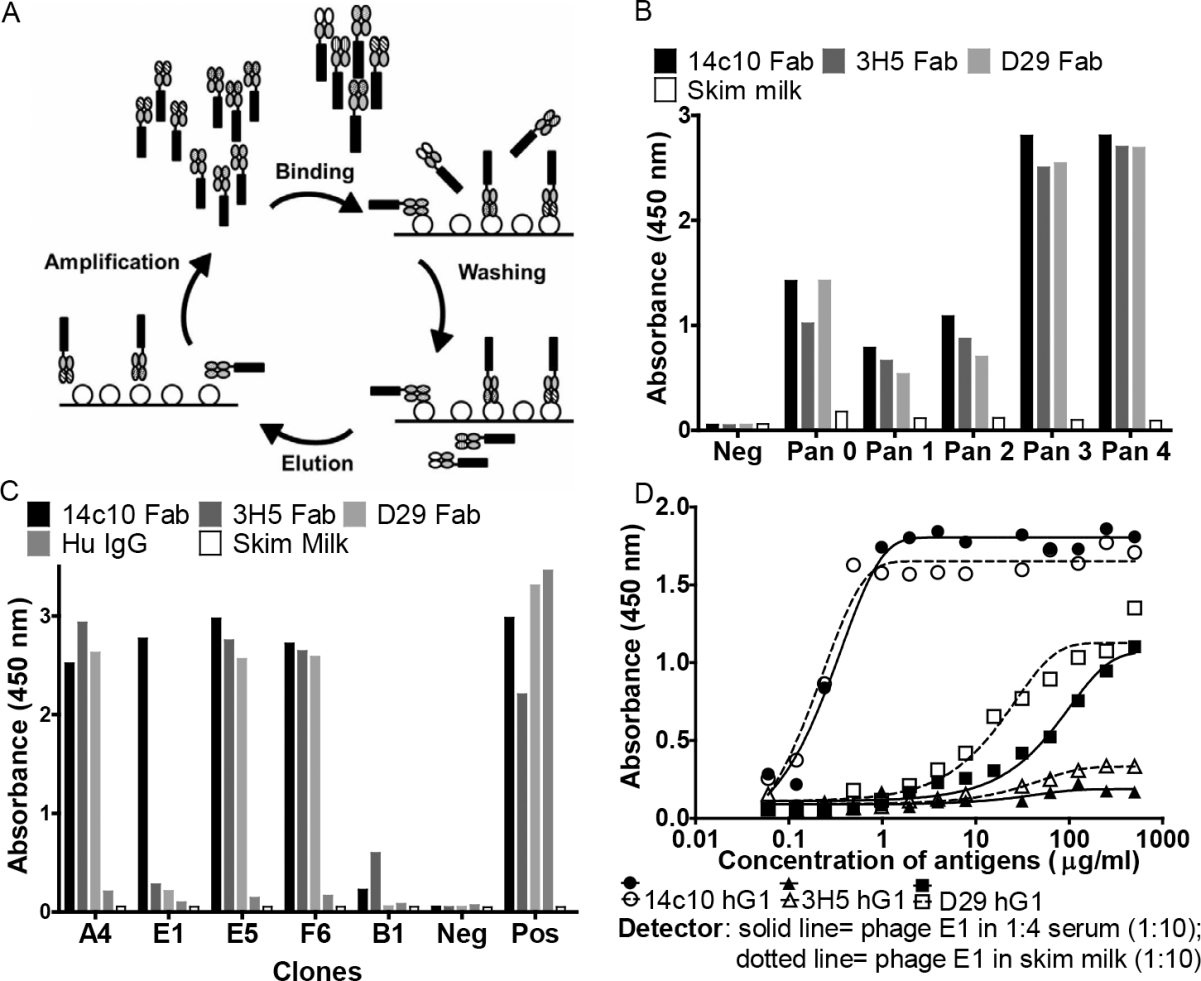
Discovery of anti-idiotypic antibody against 14c10 hG1. (A) Schematics of phage library panning which consists of four main steps; the binding of Fab expressing phage to the coated target antigen (14c10 Fab), the washing away of the unbound phage, followed by the elution and amplification of the bound phage. The cycle then repeats for the enrichment of the specific phage for the target antigen. (B) Polyclonal ELISA showing the enrichment of the successive rounds of phage panning against 14c10 Fab. The wells were coated with 14c10 Fab, other irrelevant Fabs (3H5 and D29) and blocking buffer (skim milk). Negative control was the addition of skim milk instead of the polyclonal phage to the coated antigens. Results represent one independent experiment. (C) Monoclonal ELISA showing the specificity of the monoclonal phage from pan three. Controls were included to ensure that the antigens were properly coated; negative control and positive control were the addition of skim milk instead of the monoclonal phage clones and detected with αM13- HRP (negative) and α-histag-HRP (positive) respectively. Results represent one independent experiment. (D) ELISA to test the specificity of phage clone E1 for 14c10 hG1 in serum. The antigens (14c10 hG1, 3H5 hG1 and D29 hG1) were coated. PEG precipitated phage clone E1 was used at 1 in 10 dilution either in human serum (diluted 1 in 4 with skim milk) or skim milk. Results represent one independent experiment.

A total of four rounds of panning were performed against the target antigen, 14c10 Fab with Humanyx Fab library (Humanyx Pte Ltd, Singapore) for the selection of specific Fab expressing phage (Fig 1A). The polyclonal phage from different rounds of panning was screened via ELISA against 14c10 Fab and other irrelevant antigens (3H5 and D29 Fab). These irrelevant antigens are antibodies with binding specificities to DENV, and have the same antibody scaffold as 14c10 Fab [13]. Hence, this helped us to determine the specificity of the polyclonal phage to the target antigen. In Fig 1B, we showed that there was an increased binding activity of the phage for the different antigens with increasing number of pans. This was due to the specific selection and amplification of phage for the target antigen in each round of panning. Subsequently, due to the enhanced specific binding of the polyclonal phage to 14c10 Fab, we selected the polyclonal phage from pan three for subsequent monoclonal phage selection.

From the monoclonal screening of the phage libraries (Fig 1C and S1 Fig), we found a phage clone E1 to be specific for 14c10 Fab only. In the experimental setup, we have included the positive control to confirm the presence of the coated Fab. Prior to the expression of the monoclonal phage clone E1 into a full recombinant antibody, the specificity of the clone for 14c10 hG1 in human serum was determined. The experimental setup used mimics the detection of 14c10 hG1 in serum with E1 monoclonal phage. This also allowed for the determination of the cross reactivity of E1 with other serum IgGs. In this experiment, phage clone E1 was diluted in both serum and skim milk. This was based on that if phage clone E1 binds to other serum IgGs, the binding signal to 14c10 hG1 in the presence of serum will be decreased compared to the phage clone diluted in skim milk. However, serum was used in its diluted form (four fold dilution with blocking buffer) considering its intrinsic hydrophobic nature of the polyclonal IgGs [14]. In Fig 1D, it was suggested that E1 does not cross react with other serum IgGs, as there was no reduction in the signal for 14c10 hG1 for the phage clone in serum as compared to phage diluted in blocking buffer. Hence, we expressed phage clone E1 into a full recombinant antibody.

### Expression and characterisation of monoclonal phage clone E1 in a full antibody framework

**Fig 2:**
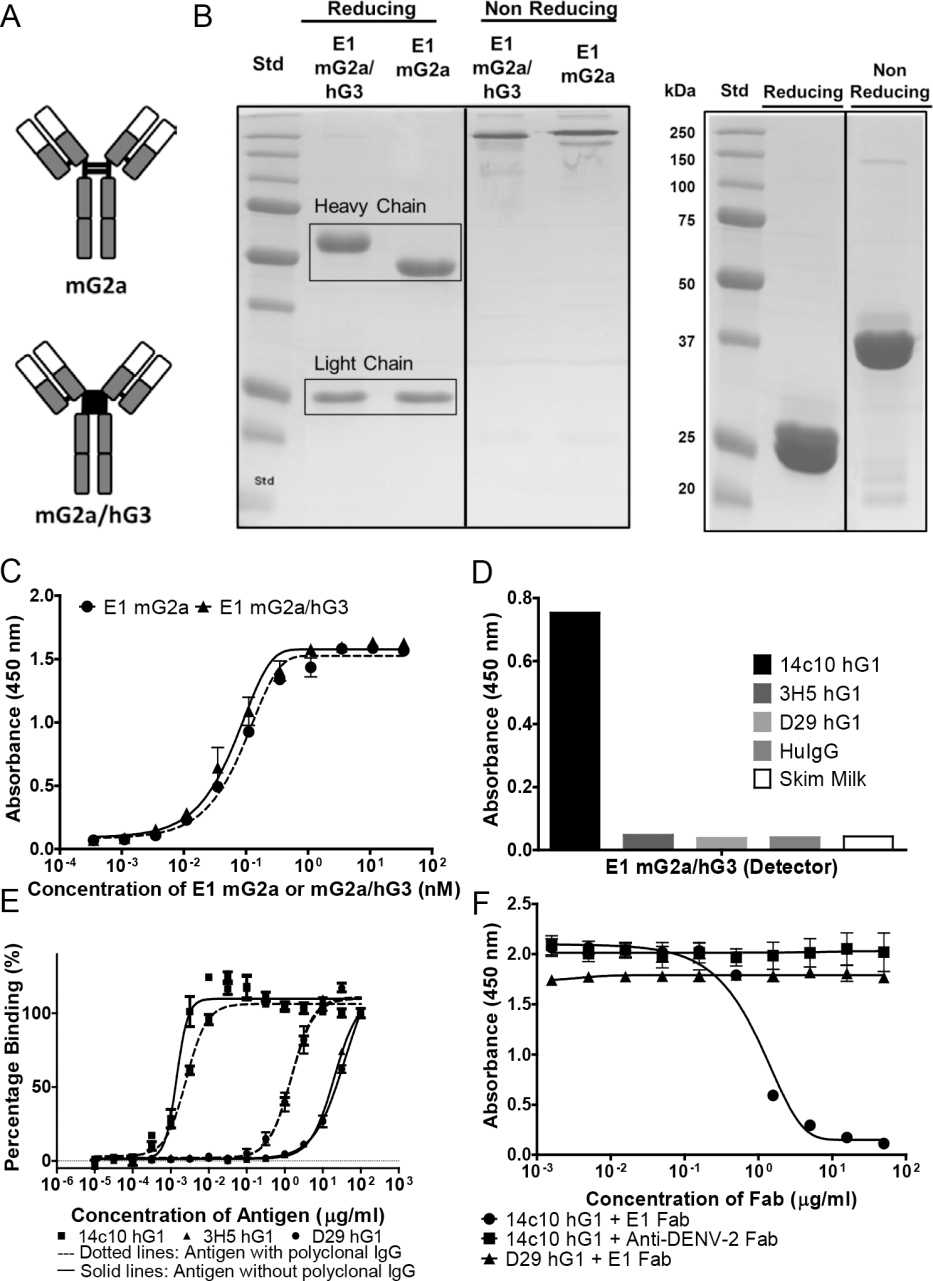
Expression and characterisation of E1. (A) Schematics showing the difference in the hinge structure between mG2a and mG2a/hG3. (B) 12% SDS-PAGE gel (reducing and non- reducing) showing the expression of E1 mG2a/hG3 and E1 mG2a (left) and E1 Fab (right). The heavy chain and light chain bands correspond to sizes, 50kDa and 25kDa respectively in the reducing gel. (C) Direct ELISA comparing the binding profile of E1 mG2a and E1 mG2a/hG3 for 14c10 hG1. The wells were coated with 14c10 hG1 and E1 mG2a and E1 mG2a/hG3 were added. Results represent average of two independent experiments performed in duplicates. Error bars represent standard error mean (SEM). (D) Determination of the binding specificity of E1 mG2a/hG3 for 14c10 hG1. The antigens (14c10 hG1, 3H5hG1, D29 hG1 and commercially available human IgG1) were coated and the detector E1 mG2a/hG3 was used, (E) the antigens (14c10/3H5/D29 hG1) with and without purified human polyclonal IgGs were added to the coated E1 mG2a/hG3. Results are representative of two independent experiments performed in duplicates. Error bars represent SEM. (F) Inhibition of binding of 14c10 hG1 to DENV-1 with E1 Fab. The wells were coated at a constant concentration with 4G2 mG2a as a capture for DENV-1. The antibodies (14c10 hG1 and D29 hG1) were used at a constant concentration and were preincubated with E1 Fab at room temperature, before the addition to DENV-1. Results are an average of two independent experiments performed in duplicates. Error bars represent SEM.

Monoclonal phage clone E1 was expressed as both mG2a and chimeric mG2a/hG3 (mouse IgG2a with human IgG3 hinge). Gel bands corresponding to 50Kda represent the heavy chain while the bands at 25kDa represent light chain (Fig 2A). As the human IgG3 hinge is larger than the mouse IgG2a hinge region (Fig 2B), the size of the heavy chain of E1 mG2a/hG3 was larger than 50kDa as compared to E1 mG2a.

We postulated that expression of the antibody in the chimeric form might increase its binding affinity to the antigen based on the flexibility of the IgG3 hinge. Hence, the binding affinities of both isotypes of E1 were tested against 14c10 hG1. From Fig 2C, we reported a slightly higher signal for chimeric E1 for 14c10 hG1 as compared to mG2a. However, there was no statistically significant difference. Nevertheless, chimeric E1 was used for the subsequent detection of 14c10 hG1 in human serum.

Next, we determined the specificity of chimeric E1 for 14c10 hG1. Other antigens such as 3H5 hG1 and D29 hG1 were included as they possess the same framework structure as 14c10 hG1 but have different variable regions. Hence, we wanted to show that chimeric E1 is specific for the variable regions of 14c10 hG1. Purified human polyclonal IgGs were also included to check the cross reactivity of chimeric E1 for them since the final utility of chimeric E1 involves measurement of 14c10 hG1 in human sera. With reference to Fig 2E, we showed that there was a high degree of specificity of chimeric E1 for 14c10 hG1. Although cross reactivity was observed at very high concentrations (100μg/ml) of other irrelevant antibodies/ antigens and at high concentration of human polyclonal IgGs, the results showed that E1 was much more sensitive for 14c10 hG1 as compared to other antibodies. The EC_50_ values of E1 mG2a/hG3 for 14c10 hG1, 3H5 hG1 and D29 hG1 (without polyclonal IgG) are 1.4ng/ml, 20.1μg/ml and 38.5μg/ml respectively. The EC50 values of 14c10 hG1, 3H5 hG1 and D29 hG1 with polyclonal IgG were 2.5ng/ml, 1.49μg/ml and 1.47μg/ml respectively (Fig 2E).

In order to deduce the binding site of E1 on 14c10 hG1, we determined if E1 was able to inhibit the binding of 14c10 hG1 to DENV-1. E1 was expressed as a Fab (Fig 2A) as its smaller size is more suitable to be used as a blocker compared to whole IgG. The size of the Fab corresponded to 25kDa in the reducing gel. E1 Fab was pre-incubated with 14c10 hG1 before the binding of 14c10 hG1 to DENV-1. At increasing concentration of E1 Fab, there was an increased inhibition of binding of 14c10 hG1 for DENV-1 (Fig 2F). The results suggest that E1 binds to the variable region of 14c10 hG1 and thus inhibits the binding of the antibody to DENV-1. We have included control antibodies (D29 hG1 and anti-DENV-2 Fab) in these experiments to show that the inhibition was a specific and true observation and not due to non-specific binding of the E1 Fab to 14c10 hG1. S2 Fig is a control experiment that demonstrates the specificities of the antibodies.

### Detection of 14c10 hG1 in serum with E1 mG2a/hG3

**Fig 3:**
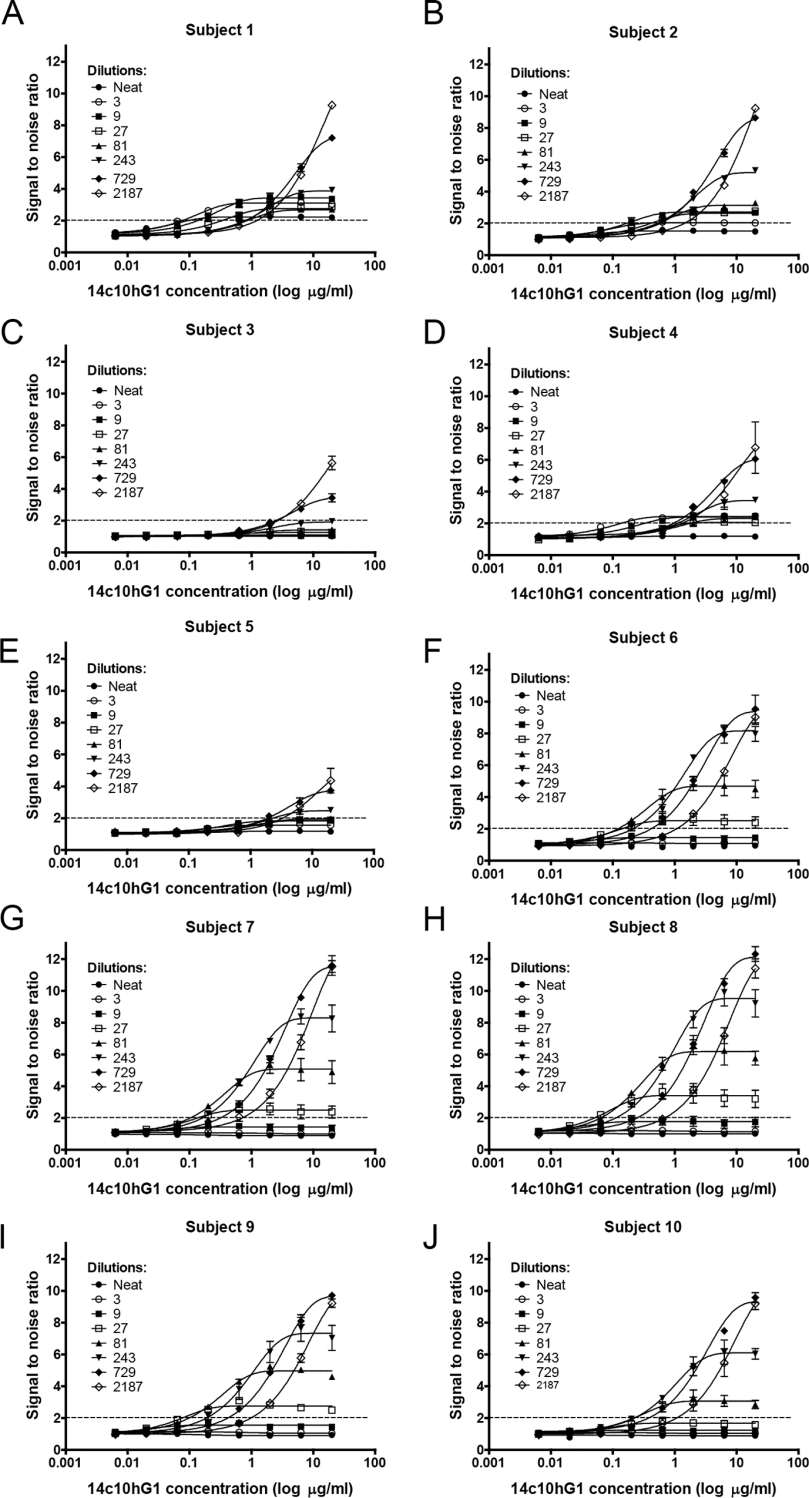
Determination of the detection limit of E1 mG2a/hG3 for 14c10 hG1 in human serum. In the sandwich ELISA assay format, the wells were coated with E1 mG2a/hG3 as a capture. Ten different healthy subjects’ sera were used at different dilutions and spiked with 14c10 hG1. Each graph is representative of the average of at least two independent experiments. Error bars represent the SEM.

The sensitivity and detection limit of chimeric E1 for 14c10 hG1 in human serum was determined using a sandwich ELISA format. The minimum limit of detection was determined based on signal to noise ratio. For the detection of 14c10 hG1, the signal to noise ratio should be at least 2. Blood sera were taken from ten different healthy human subjects to determine the limit of detection. Fig 3 and Table 1 represent the results of the detection limit of chimeric E1 for 14c10 hG1 in ten different human subjects’ serum at different dilutions. The lowest detection limit of 14c10 hG1 in human serum is 0.06μg/ml (subjects 1 and 8).

**Table 1:** Detection limit of E1 mG2a/hG3 for 14c10 hG1 in human serum was determined with ten healthy subjects’ sera at different dilutions via sandwich ELISA assay. The detection limit was determined based on signal to noise ratio larger than two. (-) denotes signal to noise ratio lesser than two. Results were representative of an average of at least two independent experiments.

	Detection Limit (14c10 hG1 concentration μg/ml)							
**Dilutions (x)**	**Neat**	**3**	**9**	**27**	**81**	**243**	**729**	**2187**
**Subject 1**	0.2	**0.06**	0.2	0.6	2	2	2	2
**Subject 2**	-	0.6	**0.2**	0.6	0.6	0.6	0.6	2
**Subject 3**	-	-	-	-	-	-	**2**	6
**Subject 4**	-	**0.2**	0.6	2	2	2	2	2
**Subject 5**	-	-	-	-	-	**2**	2	6
**Subject 6**	-	-	-	**0.2**	0.2	0.2	0.6	2
**Subject 7**	-	-	-	**0.2**	0.2	0.2	0.6	0.6
**Subject 8**	-	-	-	**0.06**	0.06	0.2	0.6	0.6
**Subject 9**	-	-	-	**0.2**	0.2	0.2	0.6	2
**Subject 10**	-	-	-	-	**0.2**	0.2	0.6	2

### Measurement of the concentration of 14c10 hG1 in AG129 mice

**Fig 4:**
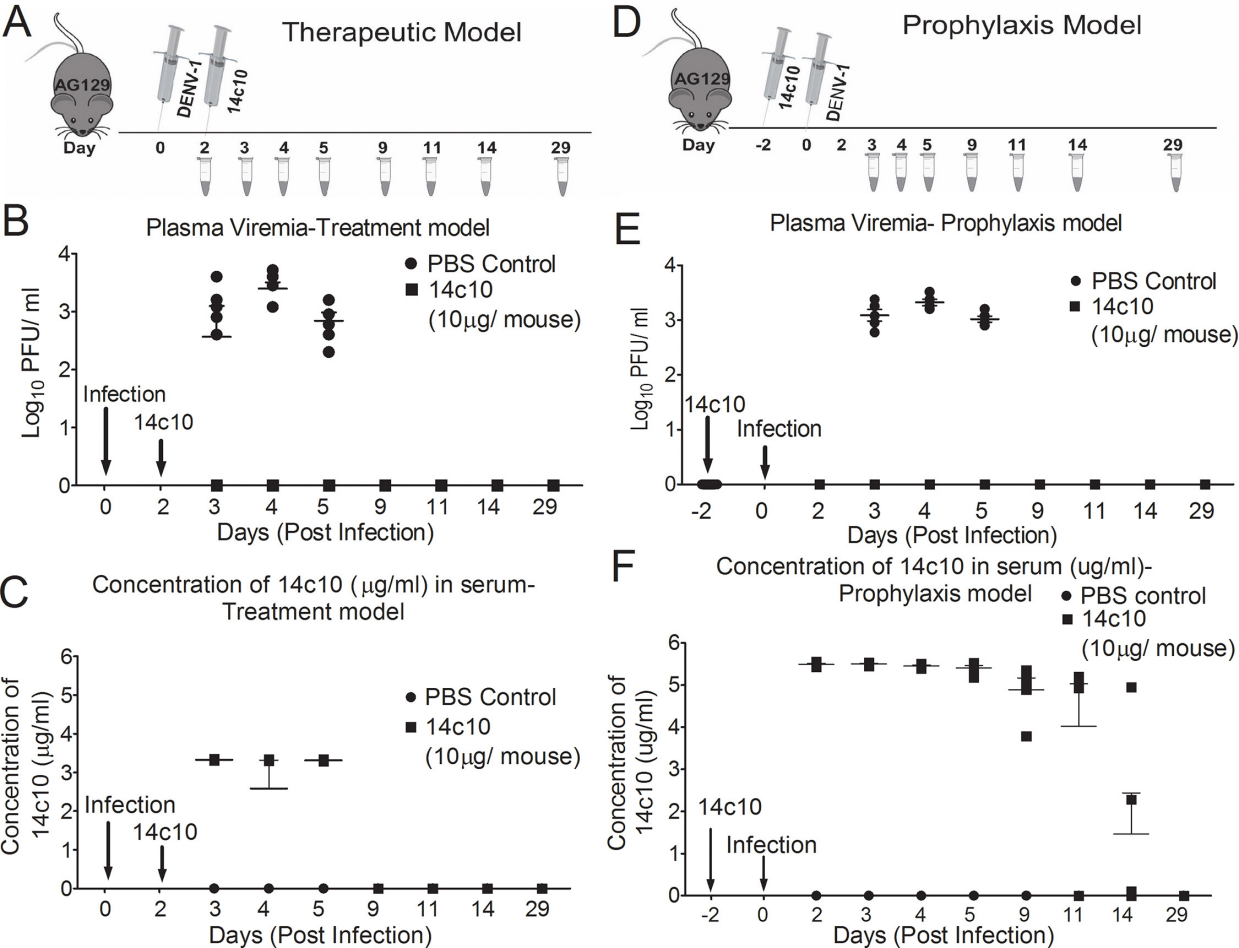
Measurement of mouse serum 14c10 hG1 with E1. AG129 mice were infected with DENV-1 WP74 (genotype 4). Ten microgram of 14c10 hG1 was inoculated intraperitoneally (i.p.) per mouse as therapy two days after infection (A- C) and as prophylaxis two days before infection (D- F). Blood was collected at various time points and the viremia level (B, E) and the concentration of serum 14c10 hG1 (C, F) was determined with plaque assay and sandwich ELISA respectively. Each group (14c10 hG1 treated and PBS control) consisted of five AG129 mice.

Besides determination of the detection limit of E1 for 14c10 hG1 *in vitro*, we also administered 14c10 hG1 into DENV-1 infected AG129 mice (therapeutic and prophylaxis models) and measured the levels of 14c10 hG1 in their serum with chimeric E1 over a period of time (Fig 4). The purpose of the experiment was to show that we are able to track the levels of 14c10 hG1 *in vivo*. These data indicated that chimeric E1 is suitable for the detection of 14c10 hG1 *in vivo* and hence for its pharmacokinetics study.

## Discussion

The initial phase in the clinical development of a therapeutic candidate antibody such as 14c10 hG1 [5], involves the determination of its pharmacokinetics (PK). For the analysis of 14c10 hG1 *in vitro* and *in vivo*, we have generated an anti-idiotype monoclonal antibody against 14c10 hG1 via the direct phage display library panning approach. Panning against 14c10 Fab allowed us to select antibodies against the relevant variable region.

The simplest and most direct way for measuring the PK of an antibody in phase I clinical studies is by direct ELISA. This involves the determination of the concentration of the antibody in the serum directly against the antigen (DENV-1). However, DENV is a biosafety level III pathogen in the United States, where the phase I clinical study will be carried out. Hence, this may not be the most efficient method for the PK measurement of 14c10 hG1. Therefore, an anti-idiotypic antibody represents an optimal reagent for the detection and measurement of 14c10 hG1 *in vivo*.

To our knowledge, anti-idiotype antibodies have not been employed previously for the analysis of fully human antibodies that have been developed as candidate anti-infectives.

Before the expression of the phage clone into a full antibody, we tested the specificity of phage clone E1 for 14c10 hG1 in serum via ELISA. However, a limitation of our experimental setup is that it might not be representative of the actual situation for the detection of 14c10 hG1 in human serum. This is because the phage was diluted in serum instead of the target antigen 14c10 hG1 being diluted in serum (which would be the situation in patients). This is because the coating of phage or serum onto ELISA plates as part of a detection methodology is confounded by the presence of a large amount of non-specific proteins [15]. Hence, our experimental set up was based on the hypothesis that any non-specific interaction of the phage with serum proteins will decrease the binding of the phage to its specific target antigen, 14c10 hG1. Based on the results we reported in Fig 1D, there was no decreased binding of the phage to 14c10 hG1. Hence, this suggests that the phage clone, E1, is specific for 14c10 hG1. Therefore E1 was expressed as a full antibody.

For the detection of 14c10 hG1 in human serum, the anti-idiotypic antibody E1 was engineered into both mG2a and mG2a/hG3 subclasses. It was previously found with another antibody that mG2a/hG3 subclass had a higher affinity for the antigen as compared to mG2a due to the presence of hG3 hinge (unpublished data). However, no significant difference was observed in the affinity of E1 mG2a and mG2a/hG3 for 14c10 hG1 (Fig 2C). Nonetheless, the subsequent experiments were carried out with E1 mG2a/hG3 due to a higher signal observed.

Before the evaluation of the detection limit of E1 for 14c10 hG1 in human serum, we first determined its detection limit in the absence of serum. The EC50 of E1 for 14c10 hG1 in the absence of serum was 1.4ng/ml (Fig 2E). In this experiment, the cross reactivity of E1 for other antibodies with the same framework as 14c10 hG1 was determined. Specificity of E1 was also tested against purified human polyclonal IgGs. This served to determine if E1 was truly specific for 14c10 hG1. We showed that cross reactivity with other antibodies such as 3H5 hG1 and polyclonal IgGs only occurs at very high concentration. Overall, there is still a significant degree of signal discrimination between the target 14c10 hG1 and other antibodies. The high binding signal observed between E1 and polyclonal IgGs could be due to genuine cross reactivity of E1 for polyclonal IgGs. This might also be due to the intrinsic hydrophobic nature of these polyclonal IgGs which might confound the true binding signal [14].

We also determined the binding site of E1 on 14c10 hG1 via ELISA (Fig 2F). We showed that E1 Fab was able to block the binding of 14c10 hG1 to DENV-1. This suggests that the site of binding of E1 on 14c10 hG1 is proximal to the variable region. In this experiment, irrelevant antibody (anti-DENV-2) was used as a control to make sure that the observed blocking of binding was true and not due to nonspecific binding at high concentration. D29 hG1 (antibody against DENV1-4) was also used in the experiment as a control to show the specific inhibition of viral binding of 14c10 hG1 by E1. Thus, we have confirmed that E1 Fab is capable of blocking the binding of 14c10 hG1 to DENV-1. However, the exact binding site of E1 on 14c10 hG1 should be further validated with a crystal structure.

The measurement assay developed for the detection of 14c10 hG1 in serum is a sandwich ELISA based assay, which make use of E1 mG2a/hG3 as a capturing antibody. The determination of the detection limit of E1 mG2a/hG3 for 14c10 hG1 was based on signal to noise ratio. In the clinical development, 14c10 hG1 will be administered to human subjects and blood will be taken from the subjects at various time points for the measurement of the concentration of 14c10 hG1 in serum. Hence, to mimic this, blood was taken from healthy human subjects, and varying concentration of 14c10 hG1 was spiked into the serum to determine the sensitivity and detection limit of E1. During the stage of assay optimisation, a small number of samples (between five to ten samples) are required [7]. Hence, blood was taken from ten healthy human subjects, and they were randomly selected based on gender, nationality and ethnicity.

The level of healthy human serum IgG is approximately 11mg/ml [16]. Thus, different dilutions of serum were tested to determine the optimal signal to noise ratio [7].

The minimum dilution is the dilution which results in a nonspecific binding signal. Hence, the assay should be able to distinguish between a true assay signal and background noise. This ensures the sensitivity of the target assay. For the detection of all clinically relevant antibodies in a clinical assay, it is recommended that the sensitivity of the assay for the measurement of antibodies in serum should be around 250ng/ml to 500ng/ml, and 500ng/ml to 1000ng/ml for preclinical studies. In addition to finding out the minimum dilution, other parameters such as incubation time, antibody concentration and number of wash cycles were also optimized [7].

Based on the results in Table 1 and Fig 3, the detection limit of E1 for 14c10 hG1 varied. The minimum detection limit was 0.06μg/ml and 2μg/ml was the detectable concentration of 14c10 hG1 in all subjects. This difference could be due to the varying specificities of IgG in the serum which might cross-react with E1 and contribute to the high serum background signal. Hence, the signal to noise ratio was affected. The varying levels and specificity of IgG in serum were dependent on the exposure of antigens in life.

Nonetheless, this detection limit is sufficient for PK studies. This is because the half- life of endogenous human IgG1 is around 23 days [17]. Based on the calculation of concentration of 14c10 hG1 at different half-lives, the assay could measure up to 4 half-lives of 14c10 hG1, which is equivalent to three months of study.

To further validate the use of E1 for the measurement of 14c10 hG1 *in vivo* (not for the purpose of pharmacokinetics study in mice), we administered 14c10 hG1 into AG129 mice in both therapeutic and prophylaxis models (Fig 4). Mice were bled at various time points to check for the levels and clearance of 14c10 hG1 with E1. With reference to Fig 4, we showed that the assay was able to determine the levels of 14c10 hG1 *in vivo*. Hence, this further ensured the validity of the assay with E1.

## Conclusion

We have generated a highly specific anti-idiotypic antibody (E1) against 14c10 hG1 with the phage display panning approach. In addition, we have developed an assay with E1 for the measurement of the concentration of 14c10 hG1 in serum, and we have validated the assay by tracking the levels of 14c10 hG1 in infected mice. The lowest possible detection limit of E1 for 14c10 hG1 in human serum was determined to be 0.06μg/ml and 2μg/ml in all subjects. This indicates that E1 can be used as an ancillary reagent for clinical studies on 14c10 hG1.

## Supporting Information

S1 Fig Determination of the specificity of monoclonal phage for 14c10 hG1. The wells were coated with antigens (14c10 Fab, D29 Fab, 3H5 Fab, HuIgG). TG1 glycerol stock containing the polyclonal phage from pan three of phage library panning was plated out and a total of 66 clones were selected. ELISA was performed on the 66 phage monoclonal clones to test their binding specificity against 14c10 Fab. Clones boxed up in black represent the positive clones for 14c10 hG1.
(TIF)

S2 Fig Controls for the inhibition of binding of 14c10 hG1 to DENV-1 with E1 Fab. The wells were coated with 4G2 mG2a as a capture for DENV-1 and DENV-2. The antibodies were used at a fixed concentration.
(TIF)
