# Supplementary figures and images for: The Molecular Engineering of an Anti-Idiotypic Antibody for Pharmacokinetic Analysis of a Fully Human Anti-Infective
Source: PLoS One. 2015 Dec 23;10(12):e0145381. doi: 10.1371/journal.pone.0145381 (PMC4689483; doi:10.1371/journal.pone.0145381)

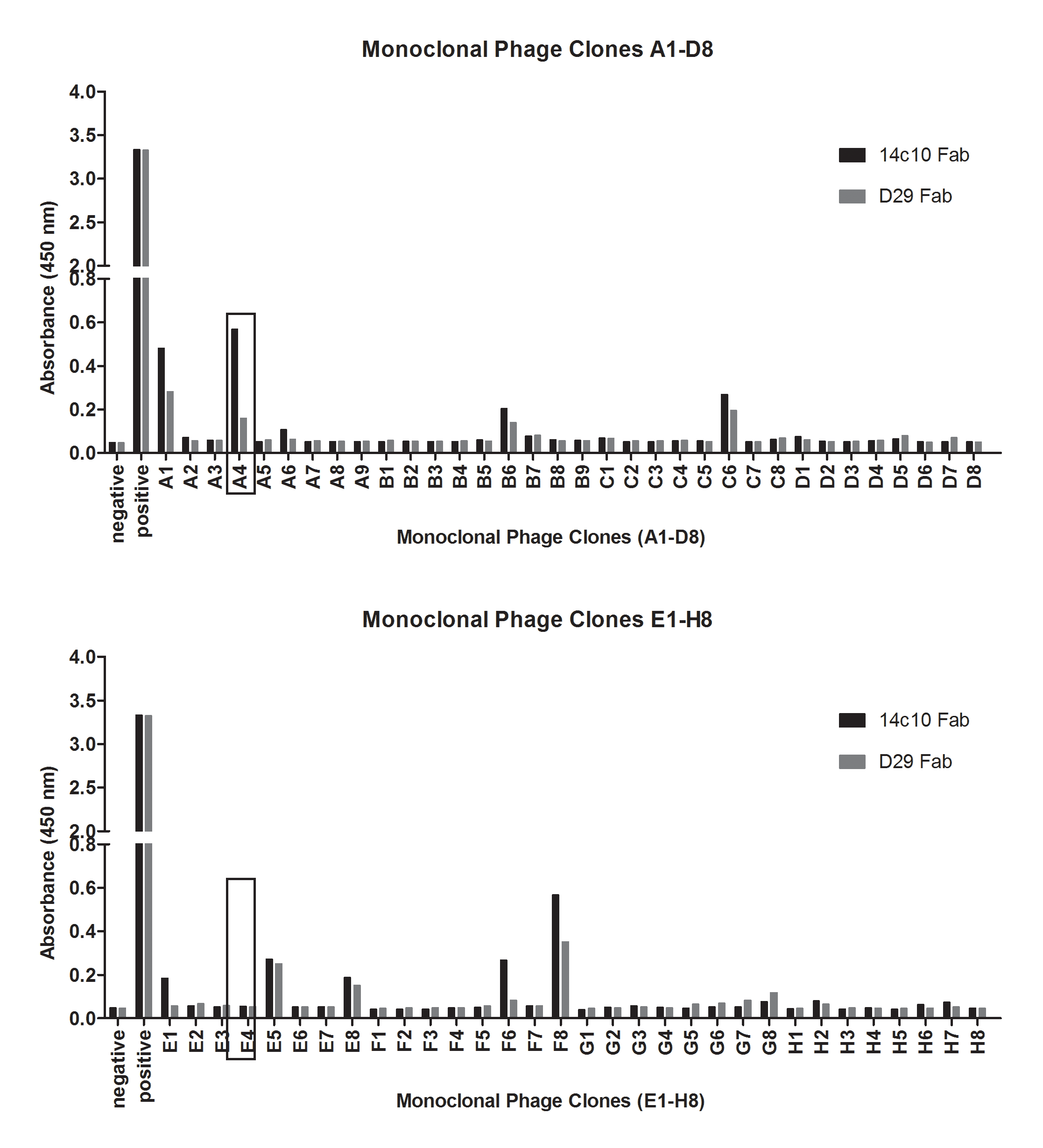

Supplement: S1 Fig — The wells were coated with antigens (14c10 Fab, D29 Fab, 3H5 Fab, HuIgG). TG1 glycerol stock containing the polyclonal phage from pan three of phage library panning was plated out and a total of 66 clones were selected. ELISA was performed on the 66 phage monoclonal clones to test their binding specificity against 14c10 Fab. Clones boxed up in black represent the positive clones for 14c10 hG1. (TIF) [file pone.0145381.s001.tif]

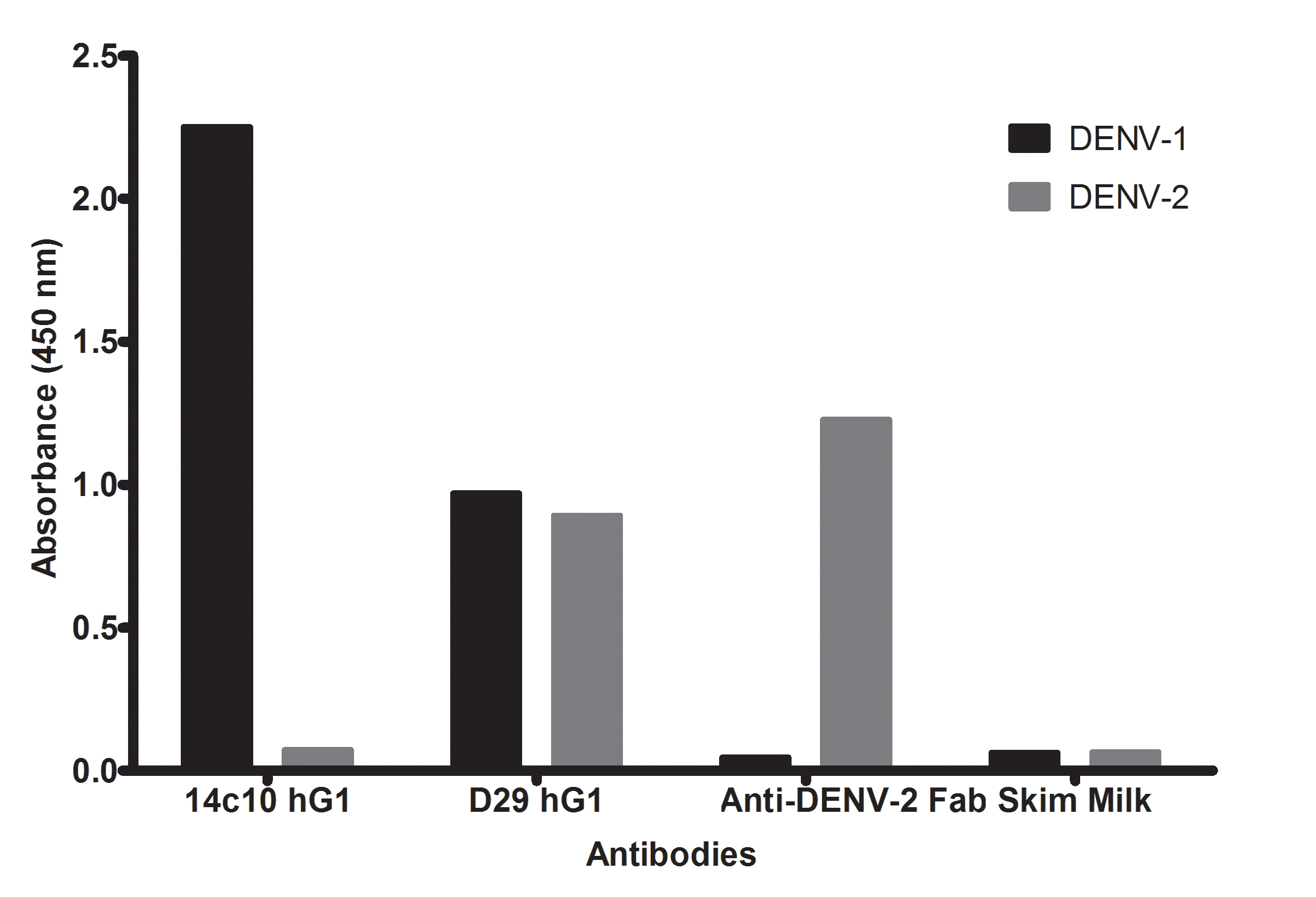

Supplement: S2 Fig — The wells were coated with 4G2 mG2a as a capture for DENV-1 and DENV-2. The antibodies were used at a fixed concentration. (TIF) [file pone.0145381.s002.tif]
